# Flexible Glass: Myth and Photonic Technology

**DOI:** 10.3390/ma18092010

**Published:** 2025-04-29

**Authors:** Giancarlo C. Righini, Maurizio Ferrari, Anna Lukowiak, Guglielmo Macrelli

**Affiliations:** 1 Nello Carrara Institute of Applied Physics (IFAC CNR), Sesto Fiorentino, 50019 Firenze, Italy; 2 Institute of Photonics and Nanotechnologies (IFN CNR, CSMFO Laboratory) and FBK Photonics Unit, Via alla Cascata 56/C, Povo, 38123 Trento, Italy; mferrari@fbk.eu; 3 Institute of Low Temperature and Structure Research, Polish Academy of Sciences, 50422 Wroclaw, Poland; a.lukowiak@intibs.pl; 4 Isoclima Group, 35042 Este, Padova, Italy; guglielmomacrelli@hotmail.com

**Keywords:** glass, flexible glass, photonic materials, solar photovoltaics

## Abstract

The recent fast advances in consumer electronics, especially in cell phones and displays, have led to the development of ultra-thin, hence flexible, glasses. Once available, such flexible glasses have proven to be of great interest and usefulness in other fields, too. Flexible photonics, for instance, has quickly taken advantage of this new material. At first sight, “flexible glass” appears to be an oxymoron. Glass is, by definition, fragile and highly breakable; its structure has puzzled scientists for decades, but it is evident that in most conditions it is a rigid material, so how can it bend? This possibility, however, has aroused the interest of artists and craftsmen since ancient times; thus, in Roman times the myth of flexible glass was born. Furthermore, the myth appeared again in the Middle Age, connected to a religious miracle. Today, however, flexible glass is no more a myth but a reality due to the fact that current technology permits us to produce micron-thick glass sheets, and any ultra-thin material can be bent. Flexibility is coming from the present capability to manufacture glass sheets at a tens of microns thickness coupled with the development of strengthening methods; it is also worth highlighting that, on the micrometric and nanometric scales, silicate glass presents plastic behavior. The most significant application area of flexible glass is consumer electronics, for the displays of smartphones and tablets, and for wearables, where flexibility and durability are crucial. Automotive and medical sectors are also gaining importance. A very relevant field, both for the market and the technological progress, is solar photovoltaics; mechanical flexibility and lightweight have allowed solar cells to evolve toward devices that possess the advantages of conformability, bendability, wearability, and moldability. The mature roll-to-roll manufacturing technology also allows for high-performance devices at a low cost. Here, a brief overview of the history of flexible glass and some examples of its application in solar photovoltaics are presented.

## 1. Introduction

**Figure 1:**
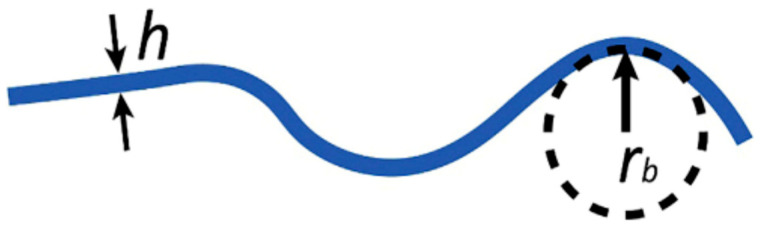
Flexibility of a material of thickness h is high when the bending radius r_b_ is small and depends on the material’s properties and its thickness. Image reproduced from [2] with the permission of Elsevier.

Flexibility limits of a material are related to its modulus of elasticity and thickness and are established by its strength or yield stress characteristics. Given the increasing requests of flexible materials in electronics and photonics, it has been suggested to use a simple figure of merit to compare different materials [1]. Let us refer to Figure 1, where a material, in sheet or wire form, with thickness h, is bent around a surface or rod with radius r_B_. The flexibility f of the material is indicated by the minimum value of r_B_ before plastic deformation (i.e., permanent deformation) occurs: f = 1/r_B_ = 2ε_y_/h [1,2]. Peng et al. proposed the simple figure of merit FOM = σ_y_/E, which is the limit or yield strain, incorporating the intensive or material properties: the yield stress σ_y_, that is, the maximum stress the material can endure before breaking or permanently deforming, and E, the Young’s (elastic) modulus [1]. Data on yield strength, Young’s modulus, flexural and bending elongation for several materials pertaining to different classes are tabled in [2]. Strength for silicate glasses is a probabilistic concept, hence the glass limit stress on a macroscopic scale σ_y_ can be considered the stress value at which glass can be exposed with a definite level of breakage probability (usually 90% or 95%).

Silicate glass is generally considered a brittle solid without plastic behavior. While this is true at a macroscopic level, on micro- and nano-metric scales it may present plastic yielding [3]. In technological applications of flexible glass, yielding effects can be neglected while strength limit shall be evaluated by looking at some modern capabilities of chemical strengthening by ion exchange [4]. Under proper conditions, ion exchange does not negatively affect the transparency of the glass, as proven by applications in integrated photonics [5]. A study on Schott AS87 glass with a thickness of 30 µm, strengthened by ion exchange, has shown that this glass exhibits plastic deformation after folding. Plastic strain recovery may also be achieved, but that depends on the loading’s history of the sample [6]. Glass plasticity could be, for instance, exploited during the fabrication process to cover curved displays or other devices.

Flexible glass, as we understand it today, is a modern invention associated with advanced technology and materials science. However, the concept of glass that bends or has some degree of flexibility has roots in ancient civilizations, where artisans were already experimenting with glass properties. Indeed, the idea of flexible glass is steeped in legend, blending history, mythology, and speculative technology. While no definitive archaeological evidence has confirmed its existence, references to ‘flexible’ glass appear in ancient writings, notably from Roman sources, and there are intriguing links to ancient Egypt’s advanced glass-making techniques. This article wishes to shed light on some of these ancient legends, still living during the Renaissance, and then to discuss some of the recent advances in the fabrication and applications of real flexible glasses.

### 1.1. Flexible Glass in Egyptian and Roman Tales

The most famous accounts of flexible glass in antiquity come from the Roman historian Pliny the Elder (AD c. 23–79) and the writer Gaius Petronius Arbiter (AD c. 27–66). Pliny the Elder authored an extensive *Natural History* in 37 books, documenting the Romans’ knowledge of the natural world. The first 10 books were published in AD 77, but Pliny’s sudden death during the eruption of Vesuvius in AD 79 prevented him from revising the remaining volumes. These were posthumously edited and published by his nephew, Pliny the Younger (AD 61–c. 113), who praised the work as “a comprehensive and learned treatise, encompassing as much as Nature herself”. In Book 36, Pliny recounts a tale of a craftsman presenting Emperor Tiberius (AD 14–37) with a cup made of flexible glass (*vitrum flexile*) [7]. When the cup was left falling to the ground, it was not broken but merely dented, and the craftsman promptly restored it with a small hammer. A scientific notation to be highlighted from a modern perspective would be “denting” and “denting repair with the hammer”. This last element suggests a plastic behavior of the invented glass, behaving like a metal with yielding properties; in some way this is more remarkable than the un-breakability condition. Tiberius, concerned that such an extraordinary material might devalue precious metals like gold and silver, allegedly ordered the artisan’s execution. Notably, Pliny approached the story with skepticism. He introduced it with the term *ferunt* (“it is said”) and concluded the narration with the phrase *eaque fama crebrior diu quam certior fuit* (“that rumor was long more widespread than reliable”).

A similar version of this story appears in *Satyricon*, a work of fiction by Petronius Arbiter: *Fuit tamen faber, qui fecit fialam vitream, quae non frangebatur* (“There was, however, an artisan who made a glass vial that could not be broken”) [8]. Like in Pliny’s account, this tale ends with the artisan’s execution rather than reward. Such narratives may have served as metaphors for the dangers that innovation may pose to established power and traditions.

The legend of flexible glass proved captivating and endured through the centuries. Petronius’s account is referenced in later works, such as *Etymologiae* (or *Origines*), an encyclopedia compiled by Isidore of Seville in the VII century, and *Policraticus* (or *Polycraticus*), an advice manual for rulers written in 1159 by John of Salisbury, a philosopher and bishop of Chartres, France. These enduring references highlight the story’s resonance as a symbol of both ingenuity and its potential perils.

An intriguing reference to flexible glass surfaced in much later writings, with connections to Egyptian history. In a book published in 1883 [9], Wallace-Dunlop mentions accounts by some Arab historians describing the activities of the Caliph Abdullah Al Ma’mun, son of Harun al-Rashid. Known for his intellect and passion for books, Al Ma’mun visited the pyramids of Giza around AD 832. Driven by a desire to uncover their secrets, he instructed his accompanying scholars to create a passage into the Great Pyramid of Khufu (Cheops). Their efforts succeeded, making Al Ma’mun the first person historically documented to enter this ancient monument. Today, visitors to the Great Pyramid still use the entrance originally opened by his team. However, according to these accounts, Al Ma’mun’s exploration was met with disappointment. Instead of the books he hoped to find, he reportedly encountered only some gold and a few jewels. Wallace-Dunlop, on the contrary, cites the Arab historian Ibn-abd-Alkhom (possibly referring to Ibn ʿAbd al-Ḥakam, an eminent Egyptian historian and jurist of the 9th century) as a source. Ibn-abd-Alkhom’s writings suggest that in the west pyramid—believed to be that of Khafre (Chephren)—great treasures were once stored, including “arms which rust not, *and glass that might be bent without breaking* (In Italic in the book by Wallace-Dunlop [9]. She also inserted the footnote ‘This is surely the first attempt at describing flexible glass’)”. While fascinating, these claims are likely intertwined with myth. Historical evidence suggests that the pyramid may have been looted during the First Intermediate Period (c. 2181–2040 BC). Additionally, another Arab historian, Ibn Abd al-Salam, dates the first recorded opening of the Khafre pyramid to AD 1372. Nevertheless, these tales fuel speculation, with rust-resistant steel and flexible glass supposedly existing over 4500 years ago. As James Gardiner wryly remarked in 1926: “Those ancients have a most exasperating way of anticipating our new ideas” [10].

The existence of flexible, unbreakable glass was also mentioned in the Kenyon Papyrus (AD 3th century) [11]. Certainly, Egypt, a hub of advanced glass-making techniques since the 18th Dynasty (c. 1550 BC), played a significant role in early glass production. Egyptians pioneered core-forming, a method used to create hollow vessels such as flasks and vases. Their mastery over glass coloration and form demonstrates their exceptional skill. Their glass, however, was typically soda-lime silica akin to modern compositions, hence inherently brittle and without the flexibility described in these legends. It appears clear that their techniques primarily aimed to imitate precious stones rather than to innovate structural properties like flexibility.

Ancient accounts of “flexible glass” might perhaps have referred to extremely thin, malleable sheets of mica or early laminates. Alternatively, they could allude to glass treated with heat or chemicals to reduce brittleness, though such methods would have been improbable in the ancient world. During Roman times, glassmakers’ techniques, including blowing and molding, produced highly refined glassware used for everyday purposes and luxury items. The properties of Roman glass often made it more resistant to shattering under mild stress compared to other materials of the time, but the notion of flexibility likely falls short of any significant measurable property resembling malleability.

### 1.2. Legend of Non-Breakable Glass in the Middle Age

In the 13th century, a new legend of unbreakable glass emerged, this time linked to a religious miracle. According to the account, a drinking glass miraculously remained intact after being thrown to the ground by a heretical knight named Aleardino da Salvaterra. This dramatic gesture formed part of a public challenge to Saint Anthony of Padua, demanding proof of his sainthood. The event is immortalized in a marble relief, crafted in 1529 and housed in the Chapel of St. Anthony within the Basilica of S. Antonio in Padua. Lucchini’s paper [12] provides a compelling analysis of this episode and other alleged glass-related miracles, offering insight into the cultural and symbolic importance of glass in medieval religious contexts.

## 2. Recent History of Flexible Glass

**Figure 2:**
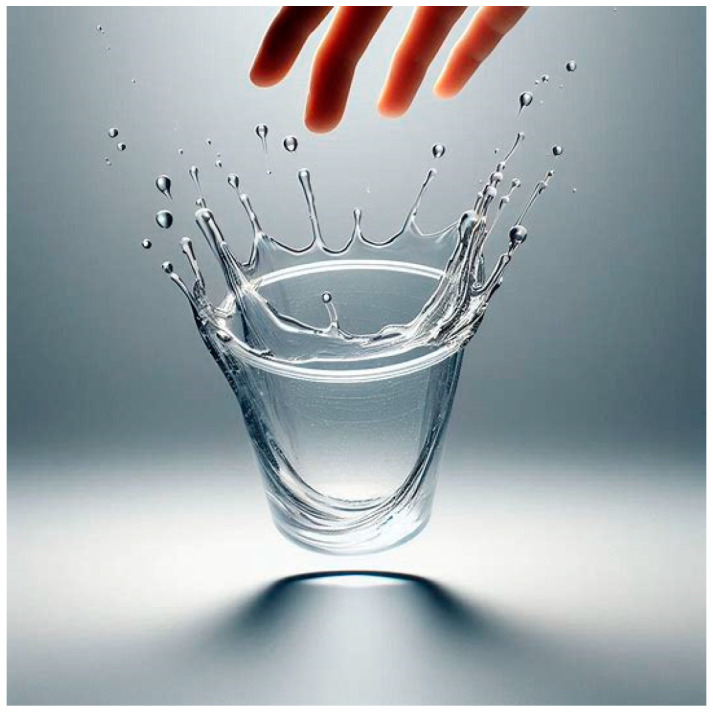
Artist’s rendering of an unbreakable transparent silicone water glass bouncing back after hitting the floor.

Although flexibility was almost certainly not a characteristic of any ancient glass, early experiments with glassblowing, casting, and layering techniques laid the foundation for later innovations. Lattermann [11] offers an intriguing review of tales and discussions from the 16th to 19th centuries about the alchemical pursuit of malleable glass. Based on the documents examined, the author deduces that alchemists interested in *vitrum malleabile* were likely searching for a material that combined two key properties: softness and deformability at room temperature or slightly higher, along with optical transparency. These properties did not necessarily align with the chemical definition of glass. A comparable result was achieved much later, with the development of silicone-based materials, which has its roots in 20th-century chemistry. Frederick Stanley Kipping (1863–1949) was an English chemist who did much of the pioneering work on silicon polymers and coined the term silicone, while in the 1930s James Franklin Hyde (1903–1999) laid the groundwork for their commercial production, which has included squishy glassware. Figure 2 shows the artist’s sketch of a silicone water glass bouncing back from the floor.

Only recently have the advances in flat glass manufacturing led to the production of high-quality, thin and ultra-thin glasses. The former definition typically refers to glass with a thickness ranging between 1.1 mm and 2 mm; actually, thin glasses production may be dated back to Roman period, but uniformly thin flat glasses started to be available only during the medieval period, and their quality and clearness were much improved in the 19th century. Nowadays, thin glass is widely used in applications like lightweight glazing, solar panels, and display screens. Ultra-thin glass (UTG), on the other hand, has a thickness of less than 1 mm, often going down to as little as 0.05 mm (50 microns) or even less; its production begun in the mid-20th century. Any ultra-thin material becomes flexible and UTG is efficiently used in advanced technologies such as foldable displays, flexible electronics, and micro-optics. More precisely, at least for discussion purposes, it is convenient to name ‘flexible’ a glass that is 200 µm or thinner. This category is not rigidly standardized but is commonly recognized across industries.

As technology progressed, especially in the development of flexible electronics, thinner glass has been engineered with enhanced properties to meet specific performance requirements. Indeed, also the pursuit of flexible glass received renewed momentum toward the end of the 20th century with the advent of flexible electronics and, later, of flexible photonics [13]. This technological shift demanded materials with a unique combination of mechanical flexibility, transparency, and durability, driving innovations that bridged the gap between ancient myths and modern scientific capabilities. Three domains, in particular, have benefited from the development of flexible devices: flexible displays, flexible integrated optics and flexible photovoltaics.

The technology of flexible displays had its origins in the mid-20th century but gained significant traction toward the late 20th and early 21st centuries. The theoretical foundations were laid in 1960s–1970s, with the research on thin-film transistors (TFTs) and organic light-emitting diodes (OLEDs): these technologies would later form the backbone of flexible displays. In 1987, at Eastman Kodak, Tang and Van Slyke built the first OLED device operated at sufficiently low voltages, paving the way for the commercialization of OLED technology [14]. Since then, research has continuously progressed, and the interested reader is referred to the rich report on the development of OLEDs in industry and of OLED emitters in academia by Hong et al. [15], as well as to the continuously updated OLED history available on the web [16]. Adding flexibility to OLEDs could create new possibilities in functionality and design of displays and other devices; indeed, it was the subject of a new wave of research. As an example, clinical applications of OLEDs in photodynamic therapy for cancer, addressing both diagnostic and therapeutic aspects, have been investigated in a very recent paper [17]. Another emerging development comes from the integration of OLED technology with quantum dots (QDs) or quantum well (QW) technologies, producing QD-OLED or QW-OLED displays, respectively [18,19]. In the former devices, OLEDs act as the light source, typically emitting blue light, which excites quantum dots, i.e., nano-sized crystalline particles that can emit light at specific wavelengths, with larger particles producing red light and smaller particles producing green light. This leads to displays with wider color gamuts and higher brightness levels, without the need for traditional color filters.

Two major types of substrates could be used for flexible OLEDs and more generally for photonic applications: polymers and ultra-thin glass. At a first glance, polymer sheets are the materials of choice for flexible substrates and for electronic circuits because they can sustain far greater strains than glass, but in general they suffer from thermal instability as well as from high permeation rates for gases and water and hence chemical stability. In contrast, standard glass is a good barrier material but is brittle and can only sustain small strains, thus strongly limiting the flexibility of the device. A solution combining chemical and mechanical resistance with flexibility was offered by the development of UTGs. The research in this field, however, had started well before many can guess. In this regard, an interesting story was told by the Japanese glass producer NSG (Nippon Sheet Glass) [20]; from around 1965, NSG began receiving numerous orders for thin glass for use in photographic plates but also wristwatches and liquid crystal panels. At the beginning, NSG produced thin flat glass ~1.2 to 1.4 mm in thickness by using the Coburn glass sheet draw process and fulfilled orders for even thinner 1.1 mm flat glass by importing it from the Belgian company Glaverbel. From around 1975, demand for wristwatch and liquid crystal panel glass grew in earnest, and NSG began development of ultra-thin flat glass. The development team succeeded in mass production in 1978 of 0.7 mm–0.55 mm soda-lime flat glass, which was named Ultra Fine Flat (UFF^®^); in the following years, the quality and yield were improved by using the float glass production process. UFF is now available in a wide range of thicknesses, from 0.28 mm up. Eventually, in 2015, mass production was initiated with a new clear chemically strengthened composition glass, named Glanova^®^, that utilized the advantages of the thin sheet float process while being low cost and stronger than conventional soda-lime glass [21].

More generally, the year 2000 saw the growth of investigations on the mechanical behavior of ultra-thin glass in view of its use as a substrate and encapsulating cover for flexible displays. One of the first published works reported, in 2002, a limited number of tests, not sufficient to perform any statistical analysis, carried out on 50 µm thick AF45 and D263^®^ Schott glasses [22]. There was some indication that thin coatings onto a UTG do improve the fracture strain. Three simulated smartcards with an embedded OLED device on 22 × 11 mm glass substrates with surface coatings all withstood 1000 cycles that produced a minimum radius of curvature of 35 mm (in the Credit card standard is 29 mm), which is equivalent to a maximum strain of 0.14%. Almost at the same time, it was also demonstrated that OLED devices with certain flexibility can be reliably built on ultra-thin glass substrates; comparison was made of devices obtained with UTG or polymeric foils [23]. In December 2003, a Schott group [24] reported the development of flexible glass substrates down to a thickness of 50 μm and showed that the deposition of an organic coating could overcome significantly the lack of mechanical stability, concluding that these flexible glass substrates were suited for production of flexible printed circuit boards (PCBs). Similar results were published two years later by another group, which showed that a UTG sheet with an optimal reinforcement polymer layer was a suitable substrate choice for flexible OLEDs/PLEDs [25]. The minimum radius of curvature of a 50 µm thick D263 glass can reach a value of 8 mm, but, due to the flaws or imperfections of the glass edges and corners, the observed minimum radius of curvature was still more than 30 mm.

At Corning, another leading glass manufacturer, the leap toward developing ultra-thin glass began in early 2007, sparked by discussions between Corning’s CEO, Wendell Weeks, and Apple’s CEO, Steve Jobs [26]. Jobs reached out to Weeks with a bold request: to create a scratch-resistant and durable glass cover for a new Apple product within just six months. Initially hesitant, Weeks knew it would be a significant challenge. Corning had been working on a highly durable glass, internally known as 0317 and later marketed as Chemcor^®^. This glass, strengthened through an ion-exchange process, could endure extraordinary bending, twisting, and pressure—up to 7 tonnes per square centimeter—but was only available in a 4 mm thickness. Apple, however, required massive quantities of glass just 1.3 mm thick—something that had never been developed or produced before. Despite the hurdles, Weeks accepted the challenge, and as a result, the first iPhone, launched in the United States on 29 June 2007, featured a Gorilla^®^ glass cover—the name given to this groundbreaking product [27].

Since then, some papers have been published dealing with the characteristics of UTGs, but the focus of R&D has been continuously moving onto their applications, which will be the subject of the next section in this paper. It shall be highlighted that, even though the strengthening of UTG by ion exchange significantly increases strength limit and allows for an extraordinary reduction in bending radius limit, significant modifications of refractive index due to the polarizability of exchanging ions impose limits in optical waveguides’ applications [4]. An excellent source of information on properties, fabrication, coating and applications of flexible glass is constituted by the book edited by S.M. Garner and published in 2017 [28]. Due to his long activity in Corning, throughout the book, Garner used Corning Willow^®^ Glass as an example of a flexible glass substrate.

On the market side, Corning surely is one of the key players. Currently, however, the global production of UTG is dominated by several leading manufacturers, each catering to diverse industries like electronics, automotive, and renewable energy. An alphabetical list of key players includes the following:

− Asahi Glass Co. (AGC) (Tokyo, Japan): Innovated ultra-thin glass for next-generation displays, including foldable screens.
− Central Glass Co., Ltd. (Tokyo, Japan): Specializes in ultra-thin glass for solar panels and electronics.
− Corning Inc. (Corning, New York, NY, USA): Renowned for its Gorilla Glass, Corning has expanded into ultra-thin glass for foldable devices and flexible electronics applications.
− Emerge Glass (New Delhi, India): A key player in the South Asian market, offering specialized ultra-thin glass products.
− Luoyang Glass Co., Ltd. (Luoyang, China): Focuses on ultra-thin glass for various industrial applications.
− Nippon Electric Glass Co., Ltd., NEG (Shiga, Japan): Produces ultra-thin glass for touchscreens and advanced display technologies.
− Nippon Sheet Glass Co., Ltd., NSG (Tokyo, Japan): Produces ultra-thin UFF and Glanova glasses widely used in the automobile industry and in the liquid crystal industry.
− Schott AG (Mainz, Germany): Offers products such as the ultra-thin Schott AS 87 Eco, designed for use in consumer electronics, particularly in smartphones and wearable devices.
− Xinyi Glass Holdings (Hong Kong, China): Supplies ultra-thin glass for electronics and the solar energy industry.

These manufacturers are at the forefront of research and development, pushing boundaries in materials engineering to meet the growing demand for flexible and durable ultra-thin glass in cutting-edge technologies. Table 1 summarizes the main technical characteristics of some of the most diffuse types of UTGs.

**Figure 3:**
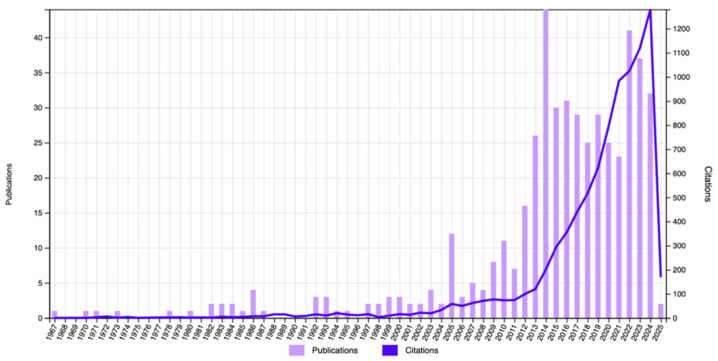
Number of publications (*y*-axis on the left) and of citations (*y*-axis on the right) having the terms ‘flexible’ <and> ‘glass’ in the title. Data from Clarivate Web of Science, accessed on 10 February 2025.

Finally, to testify the scientific interest in the characterization and application of flexible glasses, Figure 3 shows the data extracted from a search in the Clarivate Web of Science for papers containing in their title the terms ‘flexible’ <and> ‘glass’. Even if with oscillations, the growth of number of publications since the years 2000 is evident.

## 3. Photonic Applications of Flexible Glasses

Photonics is the science of light generation, manipulation, and detection. Flexible photonics, as well as flexible optoelectronics, is a rapidly advancing field at the intersection of photonics and flexible electronics [13,36,37,38], and it allows us to extend the light-managing capabilities to bendable, stretchable, or conformal platforms. It involves the integration of optical components and systems onto flexible substrates, enabling applications in areas where traditional rigid photonic devices are impractical or poorly efficient. By leveraging flexible substrates and innovative material systems, photonic devices can maintain their functionality under mechanical deformation. Two key characteristics concern waveguiding, i.e., the capability of ensuring efficient light transmission through deformable waveguides, and optoelectronic integration, i.e., the combination of flexible photonic components with electronic circuits for advanced functionalities. On the mechanical side, materials and structures must be able to withstand repeated bending or stretching without degradation.

Materials, of course, play a critical role in the development of flexible photonic devices. Durability and fabrication scalability are two main challenges. Polymers, metals and dielectrics, liquid crystals and 2D materials have all effectively contributed to the advances of flexible devices across various domains, such as photovoltaic systems [39,40], flexible displays and lighting devices [25,41,42,43], optical communication and sensing [44,45,46,47], biomedical devices and wearable technology [48,49]. Up to now, there has been no substrate suitable for all devices. In fact, the target of recent research has been to develop a wide range of materials suitable for flexible substrates, focusing on specific functionalities rather than attempting to find a substrate for all purposes. Today, various flexible substrates appear to be suitable for a broad spectrum of applications, including healthcare and human–machine interaction, and they employ non-conventional materials. Table 2 presents a few additional examples of flexible materials and applications, listed in chronological order of publication; a comparison of the properties of all the materials, however, is outside the scope of this review.

In some applications, e.g., in the fabrication of OLEDs and polymer light-emitting devices (PLEDs), the use of a UTG substrate has the great advantage of effectively protecting the organic layers from the diffusion of chemically reactive oxygen and water molecules. An early work [25] suggested to increase the robustness of the UTG and reduce the risk of cracks by depositing on it a reinforcement polymer layer; a properly diluted polydimethylsiloxane (PDMS), with the addition of aluminum 2,4-pentanedionate (C_15_H_21_AlO_6_) to control the polymer’s shrinkage, was deposited by spin coating on a 50 mm thick Schott D263 substrate. In a 1000-cycle bending test, more than ~94% of the polymer-reinforced UTG sheets passed under 30 mm or higher length compression, in comparison to only 30% of uncoated samples under the same conditions. As a conclusion, the authors claimed that polymer-reinforced UTG was suitable for fabrication of flexible or preshaped OLEDs/PLEDs. A recent work, however, has proved that, with proper processing technologies, a standard Corning Willow UTG (thickness below 300 µm) is suitable to fabricate bendable OLEDs [43]. The process is explained for a bendable OLED automotive taillight and includes several steps: bonding and debonding of the UTG to a carrier glass, UTG panel edge finishing, and UTG panel lamination on a holder. The authors also suggest that the UTG process may be used for bendable general lighting.

Due to the many different R&D fields and the large number of applications of UTG substrates, here we are forced to limit ourselves to report on the advances only in a single field, and we considered solar cells on flexible glass as a topic worth of description.

### 3.1. Flexible Solar Photovoltaic Systems

Solar cells (SCs) were among the first devices to showcase the benefits of mechanical flexibility in photonic applications. In fact, a flexible array of solar cells was proposed as early as 1967 for satellite power generation in space. This design utilized dendritic web silicon solar cells measuring 1 × 30 cm with a thickness of approximately 250 μm. The substrate was Teflon-impregnated fiberglass material, 0.0015 inch (≈38.1 µm) thick [39].

**Figure 4:**
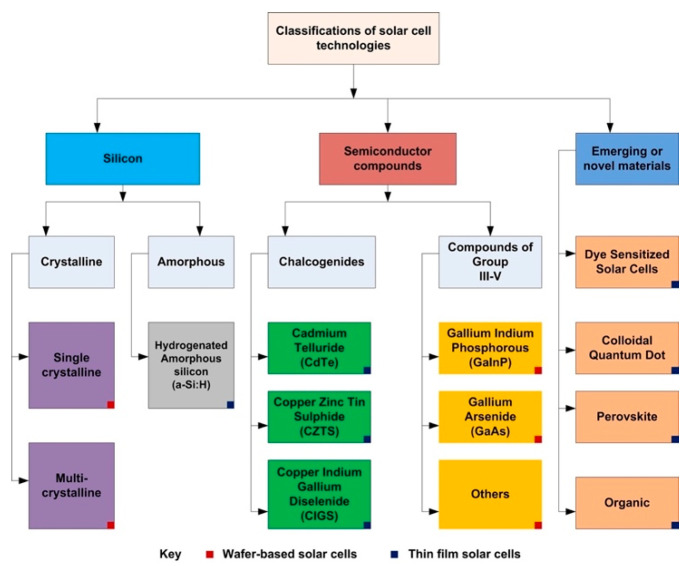
Classification of existing solar cell technologies. The structure may be wafer-based (red square in each right down corner) or thin-film (blue square). Reproduced from [60] under the CC-BY-NC-ND license.

In the last few decades, the evolution of solar cells developed incredibly fast, thanks to both a multiplication of different technologies driven by the synthesis and optimization of novel materials and a continuous increase in efficiency, made possible by proper structure design and light-management strategies [59]. Figure 4 summarizes the main types of solar cells developed so far, classified according to the active (i.e., the primary light-absorbing) material [60]. The structure of most of the thin-film SCs is based on an insulating substrate, polymers and glass being the most common.

Space applications of SCs remain of great interest even today, aboard satellites both for inner planets missions (e.g., Venus and Mars) and for travels to outer planets (e.g., Saturn and Jupiter) or into deep space; for missions lasting years, only SCs or nuclear power systems in conjunction with rechargeable batteries may guarantee uninterrupted and stable electrical power. A few excellent review papers provide an overview of the development and perspectives of solar photovoltaics in space [61,62,63,64,65,66].

**Figure 5:**
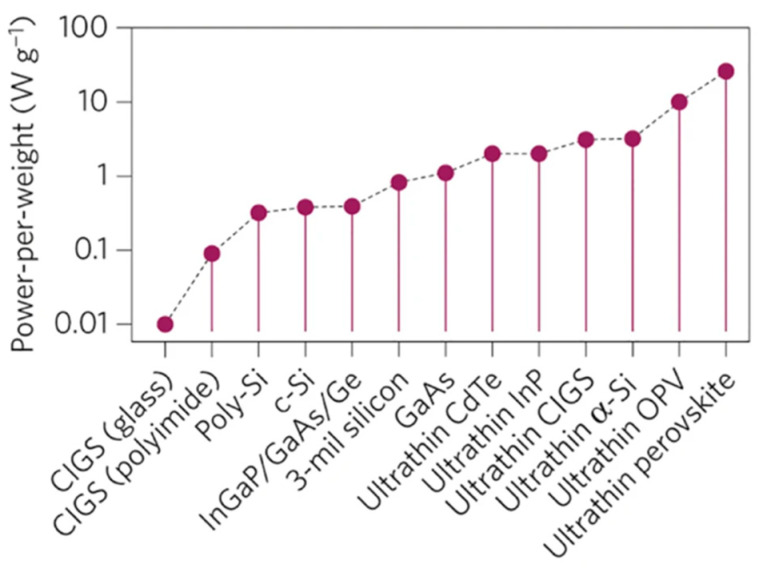
The power-per-weight of ultra-thin perovskite solar cells (UT-PSCs) is more than double the nearest competing photovoltaic technology (data extracted from publications up to 2015). Abbreviations: CIGS, copper indium gallium selenide; OPV, organic photovoltaic. Reproduced from [67] with permission of Springer Nature.

Flexible and lightweight solar cells have attracted continuously growing attention not only for use in spacecrafts and aircrafts but also for portable or wearable power sources, or on curved surfaces in building and automotive industries. Figure 5 shows how technological advances have led to the production of more and more efficient power-per-weight ultra-thin (flexible) solar cells; thanks to the high absorption coefficient, the active perovskite layer in a cell may be very thin, typically below 1 μm. In the plot, the best result refers to a perovskite solar cell (PSC), used to power aviation models, which weighs 5.2 g m^−2^ and has 3 µm thickness, including a polyethylene terephthalate (PET) substrate, electrodes and a protective encapsulating layer; it exhibits a stabilized 12% efficiency and a power-per-weight as high as 23 W g^−1^ [67].

**Figure 6:**
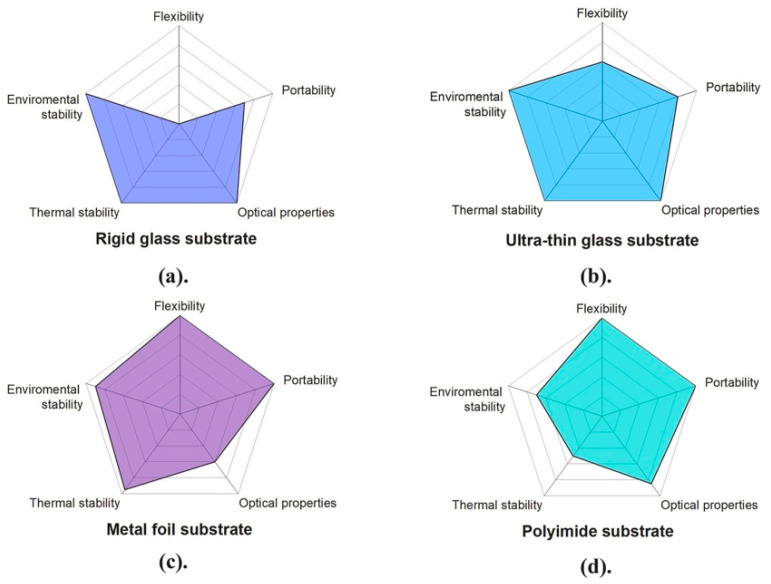
Summary of prospective substrate materials used in CdTe solar cells. (**a**) Rigid glass, (**b**) ultra-thin glass, (**c**) metal foil (highly flexible and durable, but the surface roughness affects its optical properties) and (**d**) polyimide (very light and flexible, but with limited thermal stability and resistance to moisture) substrates. Reproduced from [68] under CC-BY license.

In most of the structures considered in Figure 5, the substrate is a very thin polymeric material, but the use of UTG substrates is gaining consideration, due to their robustness, as well as their thermal and chemical resistance. Figure 6 summarizes in a graphical way the strengths and the weaknesses of the different types of substrate material, in terms of flexibility, portability, optical properties, as well as thermal and environmental stability [68]. Referring in particular to glass, which offers excellent optical and thermal properties, high mechanical strength, and durability, the rigid substrates, which possess notable chemical stability and robust resistance against environmental factors like moisture and oxidation, must face the challenges of brittleness and weight. The UTG substrates are lighter and flexible but they, too, are inherently fragile and prone to cracking or breaking under mechanical stress, so they require specialized techniques in the manufacturing and processing stages. Moreover, in terms of moisture sensitivity, ultra-thin glasses may be more vulnerable than their rigid counterparts to moisture penetration. To give a more quantitative comparison, Table 3 lists the relevant properties of a UTG flexible glass and some common flexible polymers.

**Figure 7:**
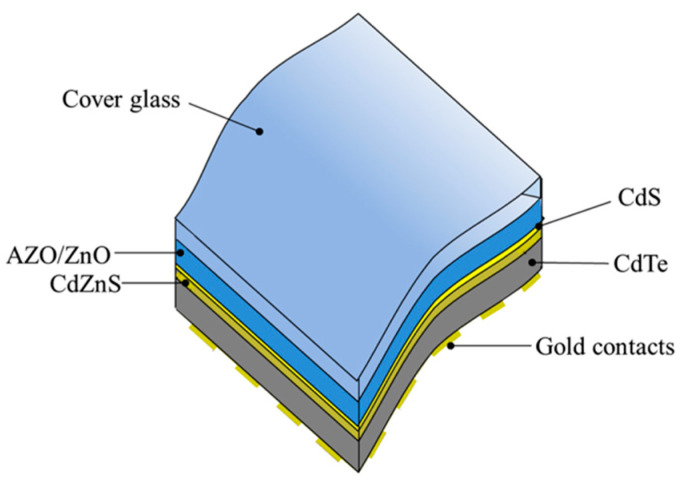
Structure of CdTe solar cell on ultra-thin (approximately 100 µm thick) cerium-doped cover glass. Underlying layers: 800 nm of Al-doped ZnO (AZO); 100 nm of undoped ZnO; 25 nm CdS seed layer; 125 nm CdZnS window layer; 3.25 μm of graded As-doped CdTe absorber layer; etched gold layer. Reproduced from [74] under CC BY license.

In the space, one of the challenges is also concerning the impact of high-energy particles, first of all electrons and protons, which change the electrical properties of the cells and cause degradation [66,72,73,74]. Glass substrates, too, are deeply affected, with a significant decrease in the transparency, particularly in the 300–600 nm range. This decrease is attributed to the creation of color centers, due to ionization-generated carriers being bound in vacancies or impurities. It is well known, however, that cerium-doped glass is very effective in absorbing high-intensity radiation that would darken any conventional glass [72], and SCs often adopt a Ce-doped glass as a protective cover. In a work by Yang et al., proton irradiation resistance of ultralightweight CdS/CdTe thin-film solar cells was investigated, and the use of ultra-thin alkali-free Corning borosilicate glass 100 µm thick was tested [73]. As expected, transmittance loss of the glass significantly increased at lower wavelengths, and a transmittance reduction of 3% was observed at 550 nm; despite this, the specific power produced by the cell after proton irradiation was still valuable and higher than the reported values of some other unirradiated SCs. An innovative solution was proposed in 2017 by Lamb et al. [74], who, using metal organic chemical vapour deposition (MOCVD), deposited thin CdTe film onto a 100 µm chemically toughened and cerium-doped cover glass, supplied by Qioptic Space Technology (now part of Excelitas Technologies Company [75]). The superstrate configuration of the cell (i.e., with the sunlight coming through the glass) is shown in Figure 7. Samples proton-irradiated at 0.5 MeV and fluence 1 × 10^12^ cm^−2^, i.e., a dose reasonably close to the value expected in a 20-year geosynchronous earth orbiting (GEO) mission, exhibited a decrease in the relative efficiency of the SC by only 5%. This excellent response was attributed to the use of Ce-doped cover UTG, which remained fully transparent whilst protecting the underlying cells from the high energy protons and electrons. The photovoltaic performance of this kind of SC was tested onboard the AlSat-1N CubeSat in low earth orbit. The data collected over some 17,000 orbits by the CubeSat in a 3-year period have shown no signs of delamination, no deterioration in short circuit current or series resistance [76].

Even if rigid silicon and III-V semiconductors still dominate the overall photovoltaic market, flexible SCs based on thin-film technologies and UTG substrates are gaining room. Let us provide some examples of promising achievements.

#### 3.1.1. CdTe Solar Cells onto UTG Substrates

CdTe must be considered a very promising solar cell material, especially for thin-film structures. In fact, it exhibits a forbidden gap of 1.45 eV very close to the maximum of solar energy conversion and, as its gap is direct, its absorption coefficient is very high, so that only a few microns of material are enough to absorb all the light at wavelengths higher than bandgap [77,78]. Thus, there has been growing interest in the development of fully flexible CdTe/CdS SCs [77,78,79,80,81,82,83,84,85,86]. An additional study of the CdTe SC based on a 100 µm thin Ce-doped glass, already mentioned in the above section [74,75,76], has shown that bending the cell with a 40 mm radius did not significantly affect the J-V characteristics of the cell [79]. Furthermore, the measurements of two cells (labeled A2 and B2, respectively) located in different positions in the 60 × 60 mm glass substrate and subjected to repeated bending did not show any degradation within experimental error. A static 32 mm bending test was performed for 168 h; the J-V was measured before and after bending at 0, 24, 48, 120, 144 and 168 h. The mean value of efficiency for all the 8 solar cells in the sample was 13.7% with a best cell performance of 14.1% for the best cell. Table 4 compares the measured values of the parameters at the initial flat state and at when the sample was reset to a flat state after the 168 h test; the performance is almost identical, within experimental error.

**Figure 8:**
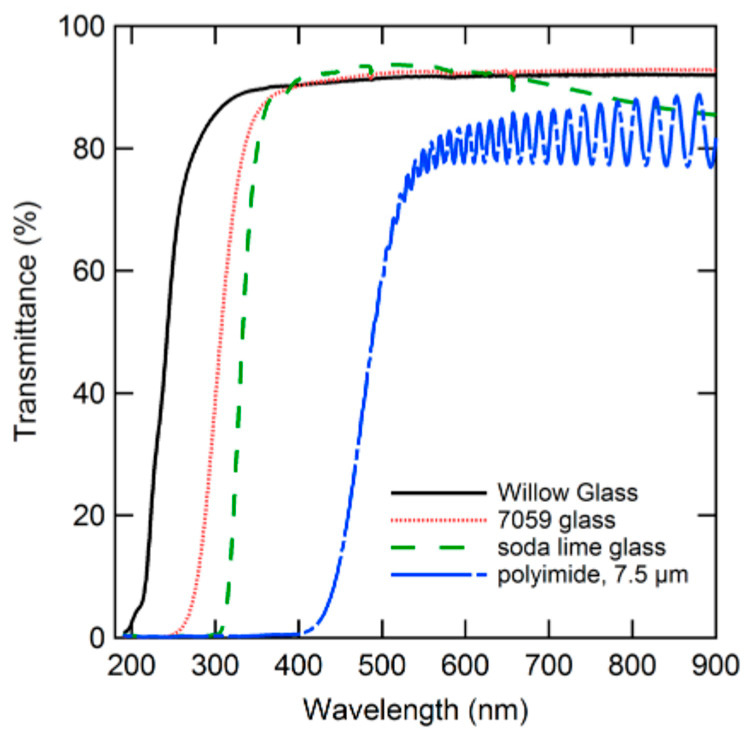
Transmittance of 100 µm thick Willow glass compared with 1.1 mm thick Corning 7059 glass, 3.8 mm soda-lime glass, and 7.5 µm thick Kapton. Reproduced from [80] with permission of AIP Publisher.

Some other papers have reported on CdTe SCs using the Corning Willow UTG as a substrate [80,81,82,85]. The excellent transmittance of Willow glass is shown in Figure 8, where it is compared with two rigid glasses (soda-lime and Corning 7059) and DuPont Kapton^®^ polyimide. Kapton has lower transparency (practically zero below 400 nm) due to the material’s absorption, whereas the lower transmission of rigid glasses below 300 nm may be attributed to their larger thickness [80].

The performance of a solar cell depends, among various factors, also on the quality of the transparent conducting oxide (TCO) used for electrical contact. TCOs are electrically conductive materials with low absorption at visible and near infra-red wavelengths; various materials are used, including fluorine doped tin oxide (FTO), indium tin oxide (ITO), and aluminum-doped zinc oxide (AZO). Liyanage et al. [82] aimed to develop an efficient process for the fabrication of CdTe SCs using as a CTO a layer of cadmium stannate (CTO); they deposited a ≈ 300 nm thick CTO layer on a 200 µm Willow glass by using reactive RF-sputtering and found that this material has excellent compatibility with the closed space sublimation (CSS) process [83,84] used for the CdTe deposition. As a result, a best efficiency of 14.4% under AM1.5 illumination was achieved with the flexible Willow glass substrate, compared with 11.7% with a rigid soda-lime substrate. A more recent paper reported an efficiency of 17.2% and 14.6% under AM1.5G and AM0, respectively (AM1.5G represents the standard spectrum at the Earth’s surface), by employing the latest advances in device fabrication. The superstrate was a 100 µm Willow glass and sputtered CTO was used as a transparent conductor [85].

**Figure 9:**
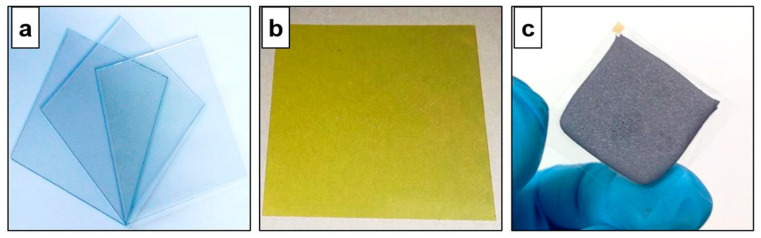
Photographs of the ultra-thin Schott D263T substrate (**a**) before deposition, (**b**) after sputtering a CdS layer ≈ 150 nm thick, and (**c**) after CSS growth of a micrometric CdTe thin film. Reproduced from [83] under CC-BY license.

The CSS deposition process of CdTe is particularly attractive due to ease in use and cost effectivity; ultra-thin glass substrates are desirable because they withstand very well the high temperatures (>500 °C) reached in the process. Doroody et al. [83] investigated the effect of the deposition temperature on the properties, e.g., grain growth and surface morphology, of CdTe thin films grown onto 100 µm thick Schott D263T flexible glass substrates. Figure 9 shows the images of a 3 × 3 cm 100 µm thick Schott D263T glass: (a) before processing, (b) after RF-sputtering of a ≈ 150 nm thick CdS layer, and (c) after CSS-deposited CdTe film (samples had thickness in the range of 6–45 µm, depending on the process temperature).

In another work, by Amin et al., an in-depth analysis is presented of the influence of pressure on the CSS growth of CdTe onto 100 µm thick Schott D263T glass [84]. In this case, the source and substrate temperature during the process were fixed at 600 °C and 500 °C, respectively, whereas different samples were processed at ambient (Argon) pressure in the range 1–200 Torr. The study demonstrated that the ultra-thin substrate did not show any deformation or structural change during the process. According to the measurements, a Torr pressure of about 1 to 5 Torr may be the optimum deposition condition for a CdTe layer on a Schott D263T substrate.

#### 3.1.2. CIGS and Perovskite Solar Cells onto Flexible Glass Substrates

Among thin-film solar cells, those based on CIGS (Copper Indium Gallium Selenide) and perovskite materials represent prominent technologies, boasting some of the highest efficiencies, often exceeding 23% in laboratories. As for the former material, the current efficiency record of 23.6% was obtained in 2024 at Uppsala University for a (Ag,Cu)(In,Ga)Se_2_ (ACIGS) composition, thanks to the introduction of a relatively high amount of silver into the absorber and the implementation of a ‘hockey stick’-like gallium profile with a high concentration of Ga close to the molybdenum back contact onto a soda-lime substrate [87]. A slightly lower efficiency of 22.2% had been achieved at Swiss Federal Laboratories for Materials Science and Technology for a flexible ACIGS solar cell with a different Ga profile (V-shaped) on a polymer substrate [88].

To be commercially competitive, the manufacturing route for a solar cell needs to be economical. The CIGS material has a direct bandgap and high absorption coefficient, so that layers as thin as 1 µm are enough to guarantee efficient sunlight absorption. Additionally, its optoelectronic properties can be tuned by varying composition in a wide range. Several solution-based techniques for CIGS, like spin coating, doctor blade technique and ink jet printing for absorber layer deposition are being widely explored; they are low-cost approaches, compared to the vacuum based sputtering and co-evaporation routes. Flexible CIGS SCs have great potential for terrestrial and space applications, and the search for proper flexible substrates instead of the widely used 0.3 mm soda-lime glass has begun quite early; one of the first approaches concerned the use of a Ti foil [89]. In a slightly later paper, 0.5–2 mil (i.e., 12.7–50.8 µm) thick Al, Ti and Mo foils were investigated as possible substrates for CuInSe_2_ layers and all three appeared compatible with the necessary selenization process, provided that a Mo layer of good mechanical integrity was interposed between the foil surface and the growing CuInSe_2_ film [90]. Various other flexible substrates such as stainless steel, polyimide and other plastics have been explored and used for CIGS flexible solar cells, but they possess inadequate thermal stability and low chemical inertness.

Since 2010, the attention has moved toward chemically inert, stable, and light-weight flexible substrates such as ultra-thin ceramics [91] and glasses [92,93,94,95,96,97,98]. CIGS solar cells grown onto 50 µm thick flexible zirconia sheets, covered by an RF-sputtered layer of soda-lime glass some 100–120 nm thick, achieved an efficiency over 16% [91]. To demonstrate the feasibility of a non-vacuum process, films made from a mixture of CIGS powder and polyethylene were applied by the doctor blade technique on Mo-coated flexible 150 µm Willow glass substrates; the films were then post-treated using an intense pulsed light (IPL) and their good quality as thin-film absorbers was confirmed by the structural and morphological characterization [92].

Looking for an ultra-thin glass with thermal stability over 550 °C and a coefficient of thermal expansion (CTE) as close as possible to the CTE of CIGS (in the range 5 to 12 10^−6^ K^−1^), to avoid cracks and adhesion problems, Gerthoffer et al. [93] choose to fabricate solar cells onto 100 µm thick Schott D263T substrates, having a CTE equal to 7.2 10^−6^ K^−1^. The 2 µm CIGS absorber layer was grown by a three-stage co-evaporation process and the 50 nm CdS buffer layer was produced by chemical bath deposition. Reference samples were also fabricated onto 1 mm thick soda-lime glass (SLG) with the same fabrication process. The best measured efficiency of the solar cell in flat state was 11.2% for the flexible device and higher than 12% for the device on SLG; this difference was attributed to the lower presence of sodium in the CIGS layer, due to the fact that there cannot be Na diffusion from the UTG, which is alkali-free. By subjecting the device to bending tests, the results indicated a significant drop in efficiency with the increase in curvature, and that performance was not recovered when resetting the device to flat state. Later, the same research group made an in-depth study of mechanical characteristics and strain of the CIGS SC on Schott D263T substrate, also measuring for the first time the CIGS hardness [94]. A relative efficiency decrease of 20% was measured after three bending cycles with a ≈50 mm radius of curvature. By comparing these results with those for a 25 µm thick polyimide substrate, it was evident that, due to smaller thickness and lower Young modulus, the latter induced less strain at a given radius of curvature.

Very recently, flexible CIGS solar cells with CdS buffer were fabricated on an 89 µm thick UTG using a well-known three-stage process, achieving a high 18.1% efficiency [97]. The authors found that the stress induced by the high temperatures (>600 °C) made the UTG substrate brittle and lose its flexibility. To avoid these problems, which are attributed to the thermal reorganization of potassium, a thin Ag precursor layer was introduced, which enabled effective grain growth at reduced temperatures (<500 °C). The optimized Ag doping (~1 at%) enhanced grain structure and device efficiency, allowing them to reach up to 17.45% efficiency at a lower temperature.

As to perovskite solar cells (PSCs), they are a relatively new class of photovoltaic technology based on hybrid organic-inorganic materials with the perovskite crystal structure (ABX_3_) [99]. Their development has been remarkably fast compared to other solar technologies: after the 2006 initial exploration, the rapid advancements since 2015 pushed lab-scale efficiencies above 25%, making PSCs competitive with traditional silicon cells. One of the main issues of perovskite SCs is stability, but great progress has also been made in this regard; a recent review paper presents the current status and the achievements in stability from 2022 to 31 July 2024 [100]. Like with other materials [101], the outstanding potential in combining the high efficiency of perovskite materials with the adaptability of flexible substrates has pushed the research for the development of flexible devices (FPSCs) [102]. UTG substrates have therefore been investigated to that purpose [103,104,105,106,107,108,109,110,111].

One of the early works concerning FPSCs used a two-step thermal evaporation method to deposit an organo-halide perovskite film onto a 50 µm thick Willow glass, obtaining a conversion efficiency up to 12.06%, which decreased by only 4% after 200 mechanical bending cycles [103].

**Figure 10:**
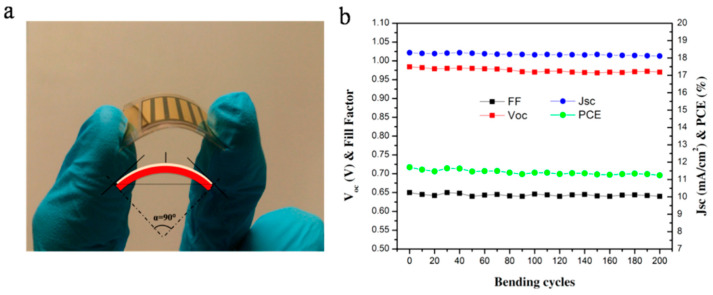
(**a**) Photograph of the flexible perovskite solar cell on a 50 µm thick Willow glass. (**b**): photovoltaic parameters (FF geometric fill factor; J_sc_ short-circuit current; V_oc_ open-circuit voltage; PCE power conversion efficiency) of the flexible perovskite solar cell (FPSC) measured after each bending cycle. Reproduced from [103] with permission of American Chemical Society.

To improve the light-trapping capability of the device, an array of nanocones was fabricated in a polydimethylsiloxane (PDMS) 0.2 mm thick film; such a structure combined anti-reflection, water-repellent and self-cleaning functions at the same time. Thanks to the strong van der Waals interaction of PDMS with glass, PDMS can be easily attached to the Willow glass. The efficiency of the flexible PSC with the nanocones increased to 13.14%. A test of bendability of the solar cells was also performed using a solar cell 3 cm long and bending it to a radius of 40 mm during 200 cycles; the SC performance was measured after each cycle [103]. Figure 10 shows, on the left, a photograph of the FPSC onto Willow glass and, on the right, the good stability of operational parameters during the test. The maximum bending angle, shown on the left side of Figure 10, was found to be 90°; beyond that, the flexible glass substrate is prone to breaking. Overall, at the end of the 200 bending cycles, the efficiency only decreased from 11.7 to 11.24%.

**Figure 11:**
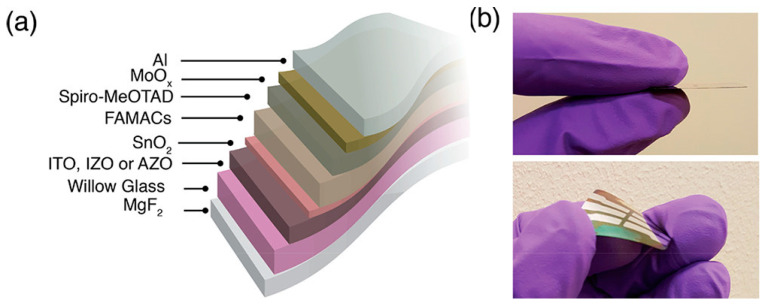
(**a**) Structure of the perovskite solar cell grown on a 100 µm thick Willow glass substrate. Different cells were fabricated using different TCOs (ITO, IZO or AZO). (**b**) Photos of the FPSC in a flat and bent status. Reprinted with permission from [106]. Copyright 2017 American Chemical Society.

A further step forward in the efficiency of an FPSC on a flexible glass substrate was made by Dou et al. [106], who optimized the cell structure by testing three different TCOs, namely, AZO, ITO and IZO (Indium Zinc Oxide). Their study demonstrated that the chemistry of the perovskite active layer can be strongly affected by the choice of the TCO on which it is grown; it came out that IZO layers are the most effective and allowed for a power conversion efficiency of over 18% to be reached. Figure 11 shows the complete structure of the FPSC under test and the photographs of the cell in a flat and bent condition.

**Figure 12:**
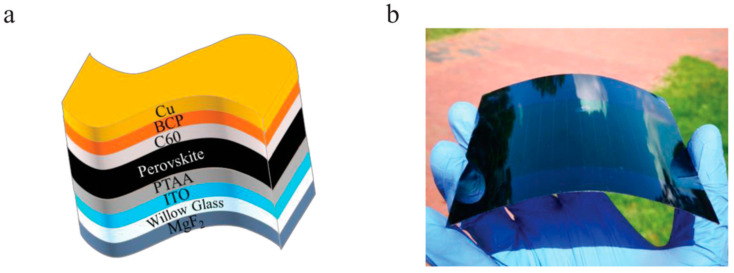
(**a**) Schematic view of the structure of a flexible large-area perovskite solar cell on Willow glass substrate. The layers from bottom up are: MgF2/Willow Glass/ITO/poly(bis(4-phenyl)(2,4,6-trimethylphenyl)amine (PTAA)/MAPbI_3_ perovskite/fullerene (C60)/bathocuproine (BCP)/copper. (**b**) Photograph of the solar module with aperture of 42.9 cm^2^ and PCE of 15.86%. Reproduced from [107] with permission of John Wiley & Sons.

Most of the reported results refer to small-area cells, often much smaller than 1 cm^2^, and it is expected that large-area cells exhibit lower power conversion efficiency due to the greater difficulty of realizing a homogeneous and densely packed perovskite film over a large area and to the series resistance of the transparent conductor electrode. An excellent performance was obtained by Dai et al., who deposited perovskite films by gas-assisted blade coating on ITO-coated Willow glass; by adding ammonium chloride (NH_4_Cl) to the precursor solution, they improved the perovskite film morphology [107]. Thus, a PCE of 15.86% was measured in photovoltaic modules with aperture of 42.9 cm^2^. A photo of the flexible module is shown in Figure 12b, whereas Figure 12a illustrates the structure of the device.

**Figure 13:**
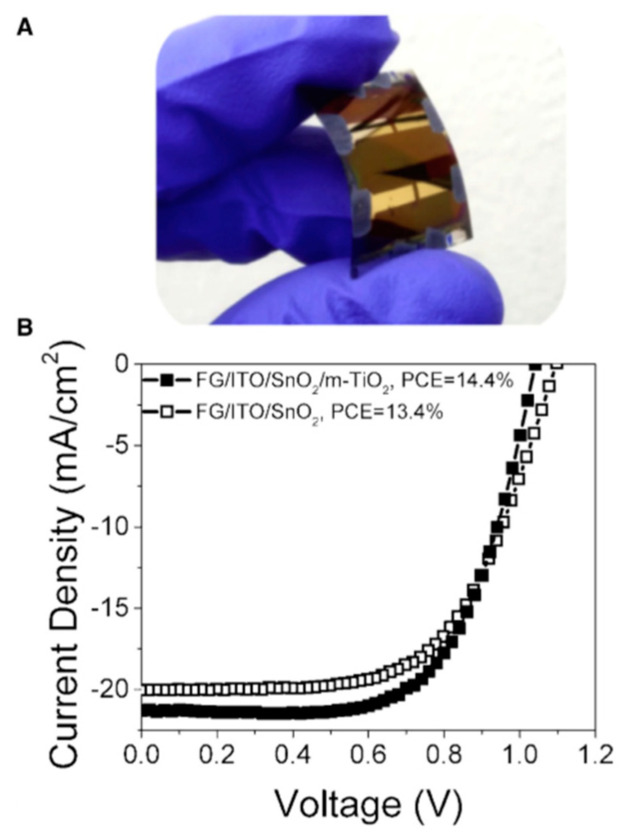
(**A**) Picture of an FPSC on 100 µm AF32 Schott glass. (**B**) J-V photovoltaic curves of planar (black squares) and mesoscopic samples at STC (white squares). Adapted from [108] under CC-BY license.

Among the applications of flexible solar cells, noticeable growth is expected for indoor devices able to supply energy sufficient for autonomous wireless sensors, low-power consumer electronics and, more generally, the internet of things system. SCs must be energy-efficient, light-weight, bendable and conformable, and flexible perovskite SCs appear to fit well such requirements. Thanks to the possibility of roll-to-roll manufacturing and processing ultra-thin glass sheets, the requirement of low cost may be fulfilled as well. Castro-Hermosa et al. reported indoor power generation by FPSCs grown on a roll of 100 µm thick AF32 Schott glass; ITO was chosen as a TCO and a ≈ 140 nm ITO layer was roll-to-roll coated on the flexible glass [108]. The other layers of the cell, except for the thermally evaporated gold contact, were also deposited in chemical-physical conditions fully compatible with roll-to-roll technology. Two types of FPSCs were fabricated and tested, which were labeled as ‘planar’ and ‘mesoscopic’, the difference being the inclusion in the latter type of ≈150 nm UV-irradiated mesoporous TiO_2_ scaffold. Both types showed high power conversion efficiency (PCE); as shown in Figure 13B, under standard test conditions (STC: 1000 W m^−2^, AM1.5G, 25 °C) the mesoporous cells exhibited slightly higher PCE (14.4%) than planar cells (13.4%).

The beneficial effect of the TiO_2_ mesoporous scaffold clearly emerged when testing the cells under indoor illumination, i.e., an OSRAM P25 white light LED. The best PCE values were 20.6% and 22.6% under 200 lux and 400 lux illuminance, respectively, for the mesoscopic sample, whereas the planar sample attained PCEs of 2.3% and 2.8%, respectively. Table 5 presents a comparison of some characteristics between the present FPSC, grown on flexible glass (FG-PSC), with perovskite solar cells grown on PET and rigid glass (labeled PET-PSC and Glass-PSC, respectively). Specific power was calculated as the ratio of power output to the weight of the solar cell, in the three illumination conditions. PET-PSC turns out to be better performing than FG-PSC only under STC, due to its lower density. Currently, PET-PSC is also more economically convenient since the estimates for industrial scale production are in the range 6–9 $/m^2^ for PET and around 40 $/m^2^ for UTG. These values, however, may change according to further innovations and market volumes.

## 4. Conclusions

The tale of *vitrum flexile* (flexible glass), as recounted by the famous Roman historian Pliny the Elder and the writer Gaius Petronius Arbiter in the AD first century, has endured through the centuries. By the 16th and 17th centuries, it remained a topic of debate. As art historian Vera Keller observes, “(In the seventeenth century) malleable glass became a prestigious scientific object. Appearing in numerous utopias, it stimulated a participatory public of scientific amateurs. Such storied objects served as vectors for spreading experimental culture, yet declined as new professions emerged” [112].

Flexible glass, long considered a utopian concept, became a reality in the 20th century when scientists—rather than artisans—demonstrated that glass could indeed be made flexible when manufactured in ultra-thin sheets and that its strength can be further increased by ion exchange processes.

By presenting a brief comparison of flexible ultra-thin glasses to other flexible, optical materials it might be noted that UTGs are characterized by relatively high flexibility, high thermal shock resistance, scratch resistance, chemical durability, and low gas permeability. Importantly, they offer high optical quality and compatibility with conventional integrated photonics systems. Polymers, on the other hand, are lighter and more flexible (have highly adaptable shapes convenient for versatile designs) but are permeable to humidity and oxygen, temperature-sensitive, and have lower efficiencies. Moreover, their manufacturing is relatively simple and cost-effective. The choice of either material is strongly dependent on application: no doubt that glass is superior in sensing applications, especially in harsh physical or chemical environments, whereas polymers may be preferable for light optoelectronic devices not subject to significant heating or when cost of the device is an issue.

Today, the advent of new flexible material substrates has propelled scientific research to new heights. The emerging field of flexible photonics marks a paradigm shift in optical system design and applications, enabling unprecedented functionalities in wearable devices, healthcare, and beyond. Emerging materials, like graphene and graphene oxide, may well contribute to further advances [113,114]; it must be noted, however, that an interesting perspective is to use graphene films onto flexible glass substrates [115,116] rather than as pure substrates. As an example, a ‘flexible graphene glass’ was synthetized by directly growing graphene on flexible glass substrates, such as Willow glass and glass fiber cloth, by means of a properly designed copper-foam-assisted plasma-enhanced chemical vapor deposition (PECVD); the produced flexible graphene glass was also used as a transparent conductive electrode for perovskite solar cells [115].

While some issues have still to be faced, advances in materials, fabrication methods, and integration strategies are steadily driving the field toward a future where photonics seamlessly integrates into everyday life.

Flexible glass’s compatibility with roll-to-roll fabrication and scalable solution-coating techniques facilitates high-throughput, high-speed manufacturing. This scalability supports a growing range of applications, from spacecraft components to wearable sensors and portable audiovisual devices. Despite ongoing challenges—such as fragility and production costs—the future of flexible glass has firmly transitioned from myth to reality.

## Figures and Tables

**Table 1:** Physical and chemical properties of some commercial ultra-thin glasses. Data are extracted from companies’ technical sheets. Numbers in square brackets, other than references, indicate samples’ thickness.

Brand Name	Unit	AGC Falcon^®^ [29]	Corning Gorilla^®^6 [30]	Corning Willow^®^ [31]	NEG G-Leaf ^TM^ [32]	NSG Glanova^®^ [33]	Schott AS87 eco [34]	Schott D263^®^ [35]
Glass type		Als	Alkali-Als	Alkaline earth Bals	Green Glass (As, Sb free)	Als	Als	Bs
Minimum COTS	µm	50	400	100	30 ± 10%	330	75	30
TL D65	%	>91.5	≥90.5 [600 µm]	>90	92 @λ = 550 nm	≥91	≥92 [330 µm]	91.7 [300 µm]
Refractive index n_d (@587.6 nm)_		1.515 ± 0.005	1.50 core 1.51 clad		1.52	1.51	1.5044 ± 0.0015	1.5231 ± 0.0015
PEC	(nm/cm)/Mpa	27.600	29.8				29	34.7
Density	g/cm^3^	≈2.48	2.40	2.56	2.46	2.48	2.46	2.51
Young’s modulus	Gpa	≈70 *	77	78.7	73	75.4	71.9	72.9
Poisson’s ratio		≈0.21 *	0.21	0.23	0.2	0.24	0.216	0.21
Shear modulus	Gpa	≈30 *	31.9					
Hardness(bct)	Kgf/mm^2^	KH 450	VH 611 (200 g load)	KH 588 (2000 g load)	VH 600	VH 528	KH 490 VH 560	KH 470 VH 510
Hardness (act)	Kgf/mm^2^	KH 546	VH 678 (200 g load)			VH 583	KH 560 VH 630	
CS	Mpa					600–800	1000 [330 µm]	290
DOL	µm					15–25		
Softening point	°C	≈665				742	855	736
Tg	°C	≈575				554	598	557
Strain point	°C			725		508	577	529
Annealing point	°C			781		552	616	557
CTE × 10^−7^	/°C	≈90 25–300 °C	75.2 0–300 °C	31.7 0–300 °C		91.8 50–350 °C	92 20–300 °C	
Thermal conductivity	W/(m × K)	≈1.19 *						
Dielectric constant					5.3 (1 MHz, 25 °C)		8.4 (1 MHz, 25 °C)	6.7 (1 MHz, 25 °C)
Notes		♣	♦	♥				
Suggested applications	See notes:	{A}	{B}	{C}	{D}	{E}	{F}	{G}

Abbreviations: Als, aluminosilicate; Bals, boro-alluminosilicate; Bs, borosilicate; COTS, commercial off the shelf; TL D65, light transmission indicating the percentage of sunlight passing through the glass, where D65 refers to the illuminant according to DIN 67 507; cl, compression layer; PEC, photoelastic constant; bct, before chemical strengthening by ion-exchange; act, after chemical strengthening by ion-exchange; KH, Knoop hardness; VH, Vickers hardness; CS, compressive stress (surface compressive stress); DOL, depth of layer; Tg, transformation temperature; CTE, coefficient of thermal expansion; * indicates computed values for AGC Falcon. Notes: ♣ AGC in 2014 claimed to have successfully rolled ultra-thin sheet glass SPOOL^TM^ into a roll 1150 mm wide and 100 m long, with 50 µm thickness; at that time, the world’s thinnest glass was manufactured using the float process. ♦ In Corning lab tests, Gorilla Glass 6 survived drops from up to 1.6 m onto hard, rough surfaces. That was the average worldwide height. ♥ Corning is also producing a Willow glass based on an alkali-free borosilicate. Here, the indications by manufacturers on possible applications of their products are reported: {A} Electronics (smartphones, tablets, laptops, interactive displays, et.); transportation (trains, aerospace, automotive, etc. (interior and exterior)); building (lightweight assemblies, creative designs, etc.). {B} Ideal protective cover material for the front and back of all electronic devices: smartphones, notebook PCs, tablets, smartwatches and wearables, smart home devices, cameras, commercial and point of sale displays. {C} Willow Glass is also used in architectural applications to provide a high-gloss, durable surface finish that can withstand the effects of commercial cleaning agents. {D} G-Leaf is a next-generation material that holds excellent potential for applications such as electronics, energy-related products, medical-use products, and lighting. {E} Glanova is an ultra-thin sheet glass for chemical strengthening that is ideal for use as cover glass for automotive CIDs, clusters, and electronic devices. {F} AS 87-neo meets the high demands of modern high-performance technology for robustness, offering exceptional impact and bending strength. The specialty glass enables foldable, well-protected devices without compromising touch sensitivity or functionality. {G} Schott D 263 performs consistently well in demanding conditions. Its high chemical resistance makes it particularly resilient in the consumer electronics, semiconductor and biotech fields. Variants of D 263 focus on specific requirements in particular applications. D 263 bio is suited to medical diagnostics, while D 263 M provides accurate microscopy cover slips. Schott D 263 T eco is used in imaging, sensing and semiconductors, as well as RF/HF applications, and D 263 LA eco is effective in image sensor systems.

**Table 2:** Examples of photonic applications exploiting flexible materials.

Year	Material	Application	Ref.
2013	Corning Willow UTG	Electrophosphorescent OLED	[50]
2017	Gold nanorods onto SU-8	Conformable holographic metasurfaces	[51]
2017	Chalcogenide glass	Flexible integrated optical circuits	[52]
2019	III-Nitride nanowires onto metal foils	Flexible integrated photonics (LEDs and lasers)	[53]
2021	MEH:PPV polymer	Single-layer OLED	[54]
2021	Perovskite	Solar cell	[55]
2023	Carbon- and fiber-reinforced polymers	Flexible integrated photonics	[56]
2025	Polyethylene naphthalate (PEN)	Flexible electroluminescent devices	[57]
2025	AgNps-coated polyamide	SERS detectors of hazardous substances on curved surfaces for food safety	[58]

Notes: SU-8 is a commercial, high-contrast, epoxy-based photoresist; MEH:PPV: Poly [2-methoxy-5(2′-ethylhexyloxy)-1, 4-phenylenevinylene; SERS: surface-enhanced Raman scattering; AgNps: silver nanoparticles.

**Table 3:** Mechanical, thermal and optical characteristics of a flexible glass and three of the most widely used polymers.

Property	Flexible Glass	PET [69]	PEN [70]	PDMS [71]
Young’s Modulus (GPa)	70–75	2–4	4–6	0.001–0.01
Tensile Strength (MPa)	200–500	55–75	100–150	~2
Elongation at Break (%)	<1	100–300	100–200	~100
CTE (×10^−6^/°C)	3–7	50–70	20–40	~300
Thermal Conductivity (W/m·K)	~1.0	0.15–0.24	0.2	0.15
Maximum Service Temperature (°C)	>500	150–200	200–250	200–300
Glass Transition Temperature (°C)	~550	67–81	120–155	~125
Density (g/cm^3^)	~2.4	1.38	1.36	0.97–1.05
Optical Transparency (%)	>90	~90	~89	95–98

Notes: The data for the flexible glass refer to Corning Willow glass. PET: polyethylene terephthalate; PEN: polyethylene naphthalate; PDMS: polydimethylsiloxane; CTE: coefficient of thermal expansion.

**Table 4:** Measured operational parameters of two solar cells in a flat state before (t = 0) and after (t = 168 h) bending tests. Here, η is conversion efficiency, J_sc_ is short-circuit current density, V_oc_ is open circuit voltage, FF is fill factor, R_s_ is series resistance and R_shunt_ is shunt resistance. Data taken from [79] under CC-BY license.

Solar Cell	Time (h)	η (%)	J_sc_ (mA/cm^2)^	V_oc_ (mV)	FF (%)	R_s_ (Ω cm^2^)	R_shunt_ (Ω cm^2^)
A2	0	14.1 ± 0.3	25.4	747	74	2.4	3233
	168	13.8 ± 0.3	24.7	745	75	2.4	3254
B2	0	14.1 ± 0.3	25.4	752	74	2.4	3627
	168	14.2 ± 0.3	25.7	751	74	2.5	5122

**Table 5:** Comparison of some parameters for perovskite solar cells (PSCs) fabricated on flexible glass, PET, and rigid glass. Reproduced from [108] under CC-BY license.

	Specific Power (W g^−1^)			Area Density (g/cm^2^)	Thickness (µm)
	STC	400 lx	200 lx		
FG-PSC	0.58	1.4 × 10^−3^	0.7 × 10^−3^	251	100
PET-PSC	0.74	0.9 × 10^−3^	0.5 × 10^−3^	198	125
Glass-PSC	0.07	1.5 × 10^−4^	0.7 × 10^−3^	2761	1100
